# Clinical value of IL-17-targeted intervention in tissue injury repair: from bidirectional mechanisms to therapeutic strategies

**DOI:** 10.3389/fimmu.2026.1773504

**Published:** 2026-06-02

**Authors:** Wenyu An, Xinyi Ma, Runxue Guan, Yufen Zhao, Nina Yang, Liang Jin, Yang Jian, Shaoying Gao, Zairong Wei

**Affiliations:** 1 Department of Burns and Plastic Surgery, Affiliated Hospital of Zunyi Medical University, Zunyi, Guizhou, China; 2 The 2011 Collaborative Innovation Center of Tissue Damage Repair and Regeneration Medicine, Affiliated Hospital of Zunyi Medical University, Zunyi, Guizhou, China; 3 Emergency Intensive Care Unit, Affiliated Hospital of Zunyi Medical University, Zunyi, Guizhou, China; 4 Guizhou Biofabrication Laboratory, Affiliated Hospital of Zunyi Medical University, Zunyi, Guizhou, China

**Keywords:** fibrosis, IL-17, inflammatory response, Th17 cell, tissue repair

## Abstract

IL-17, a pleiotropic cytokine, activates downstream signaling pathways through the IL-17 receptor (IL-17R), influencing the expression and regulation of inflammatory mediators, growth factors, and matrix metalloproteinases, thereby modulating various biological processes. Recent studies have shown that IL-17 exhibits dynamic biphasic effects during tissue repair: in the acute phase, it accelerates tissue repair by promoting epithelial regeneration, angiogenesis, and the recruitment of reparative immune cells, whereas in the chronic phase, excessive activation leads to uncontrolled inflammation and fibrosis progression. This “double-edged sword” effect shows significant heterogeneity across different tissues, including the skin, lungs, and intestines. Despite the widely recognized dual roles of IL-17, the field still lacks a holistic perspective that systematically explains how this duality is regulated across different tissue environments. This review outlines recent advances in IL-17’s functional dichotomy and identifies cellular origin, injury phase, and local microenvironment as critical regulatory determinants. We aim to elucidate under what circumstances and why IL-17 promotes tissue repair rather than exacerbates fibrosis, and discuss the implications of these insights for developing therapeutic strategies tailored to distinct clinical scenarios.

## 1 Introduction

Tissue injury repair is a highly coordinated pathophysiological process involving multiple stages, including inflammation, cell proliferation and migration, angiogenesis, and extracellular matrix remodeling (1). In recent years, the IL-17 family (especially IL-17A and IL-17F) has been identified as a key regulatory molecule linking immune responses to tissue repair (2, 3). It is primarily secreted by Th17 cells, γδ T cells, and type 3 innate lymphoid cells (ILC3) and activates downstream signaling pathways such as NF-κB, MAPK, JAK/STAT, and C/EBPβ via the IL-17R/Act1/TRAF6 signaling axis. This, in turn, regulates the expression of inflammatory mediators, growth factors, and matrix metalloproteinases, thereby maintaining the inflammation-regeneration balance in the injury microenvironment (4). Traditionally, IL-17 has been extensively studied for its central role in host defense and autoimmune diseases such as psoriasis and rheumatoid arthritis (5, 6).

With advancing research, a growing body of evidence has revealed that IL-17 can both promote and hinder tissue repair depending on the pathological context (2, 7, 8). While IL-17 is well recognized as a double-edged sword, current evidence remains largely organ-specific and fragmented, with apparent contradictions that have not been systematically resolved. This review provides an integrated synthesis across multiple organ systems, focusing on common determinants—cellular source, injury phase, signaling intensity, and microbial context—that govern the protective versus pathogenic switch of IL-17. We clarify when and why IL-17 promotes tissue repair rather than fibrosis, and discuss the implications for clinically tailored therapeutic strategies.

To ensure transparency and rigor in this narrative review, we conducted a comprehensive literature search using the PubMed, Web of Science, and Scopus databases. The search covered the period from January 2012 to April 2026, with the year 2012 selected as a starting point because it marked the publication of a seminal review by Miossec and Kolls in *Nature Reviews Drug Discovery* that firmly established the therapeutic potential of targeting IL-17 in chronic inflammation. Search terms included “IL-17,” “interleukin-17,” “IL-17A,” “IL-17F,” “Th17 cells,” “γδ T cells,” “tissue repair,” “wound healing,” “regeneration,” “fibrosis,” “inflammation,” and specific organ terms such as “skin,” “lung,” “intestine,” “liver,” and “muscle.”

## 2 Phases of tissue injury repair

Tissue injury repair is a highly coordinated and dynamic process involving four overlapping stages: hemostasis, inflammation, proliferation, and remodeling, each precisely regulated by specific cellular and molecular events (9).

The hemostasis phase marks the initiation of repair, characterized by vasoconstriction, platelet activation, and the activation of the coagulation cascade. Platelets aggregate to form a temporary plug and recruit inflammatory cells through the release of growth factors such as PDGF and TGF-β. Additionally, the formation of a fibrin mesh not only stabilizes the clot but also provides a scaffold for subsequent cell migration (10).

During the inflammation phase, neutrophils rapidly infiltrate the injury site, clearing necrotic tissue and pathogens through the release of reactive oxygen species (ROS) and matrix metalloproteinases (MMP-8/9), thereby playing a pivotal role in early defense. Subsequently, monocytes differentiate into macrophages, transitioning from a pro-inflammatory (M1) to an anti-inflammatory/reparative (M2) phenotype, thereby promoting inflammation resolution and secreting growth factors such as Vascular Endothelial Growth Factor (VEGF) and Fibroblast Growth Factor (FGF), which create conditions for tissue regeneration (11, 12). However, it is important to note that excessive or prolonged inflammation can lead to chronic, non-healing wounds or exacerbate pre-existing infections. In contrast, insufficient inflammation may impair tissue barrier and immune defense functions, reduce debridement efficiency, and delay the repair process (13, 14).

In the proliferation phase, the focus of repair shifts toward the reconstruction of tissue structure and the promotion of functional recovery. Epithelial cells at the injury margins, such as keratinocytes and alveolar epithelial cells, repair the damaged areas through migration and proliferation. For cutaneous keratinocytes, this process is driven by growth factors and cytokines that activate migratory and proliferative programs (15–19). During the proliferative phase of lung repair, alveolar epithelial regeneration depends on multiple immune subsets (e.g., Tregs, macrophages, CD8^+^ T cells) secreting key factors such as KGF and IFN−γ to coordinate epithelial proliferation (20–22). Meanwhile, fibroblasts become activated and secrete type III collagen to form a highly vascularized granulation tissue, facilitating healing (23–25). Additionally, during this phase, factors such as VEGF and angiopoietin significantly increase, promoting angiogenesis and providing nutritional and oxygen support to the regenerating tissue. However, dysregulation in this phase can lead to repair stagnation or abnormal proliferation, such as the formation of hypertrophic scars (26, 27).

In the remodeling phase, which can last from weeks to months, extracellular matrix metalloproteinases and their inhibitors (TIMPs) regulate matrix degradation, while myofibroblasts contribute to wound closure through contraction. However, if myofibroblasts remain persistently activated, pathological fibrosis may occur, leading to conditions such as keloids or pulmonary fibrosis (28–30).

## 3 The IL-17 family and IL-17 signaling pathways

The IL-17 cytokine family comprises six structurally related members (IL-17A to IL-17F), which play pivotal roles in immune defense and the maintenance of tissue homeostasis. Among them, IL-17A (commonly referred to as IL-17) and IL-17F are the most representative members, primarily produced by activated Th17 cells, γδ T cells, and ILC3. In contrast, other family members have distinct cellular origins; IL-17B is derived from Schwann cells and acts in an autocrine manner via IL-17 receptor B (IL-17RB) to promote macrophage recruitment and myelin clearance (31); IL-17D, produced by type II alveolar epithelial cells, contributes to the development and progression of lung cancer and acute lung injury (32, 33). IL-17E (also called IL-25) is produced by epithelial cells and Th2 cells, and is strongly linked to type 2 immunity (34); and IL-17C is predominantly produced by epithelial cells at barrier surfaces, where it acts in an autocrine manner to regulate innate immunity and tissue homeostasis (35–37). These cytokines initiate downstream signal transduction by binding to a cell-surface receptor complex composed of IL-17RA and IL-17RC subunits. Importantly, the binding affinities of different IL-17 family members to their receptors vary considerably: IL-17A displays the strongest affinity, followed by the IL-17A/F heterodimer, whereas IL-17F exhibits relatively weaker binding affinity. This gradient of receptor-binding affinity provides a molecular basis for the fine-tuned regulation of immune response intensity *in vivo* (38).

**Figure 1:**
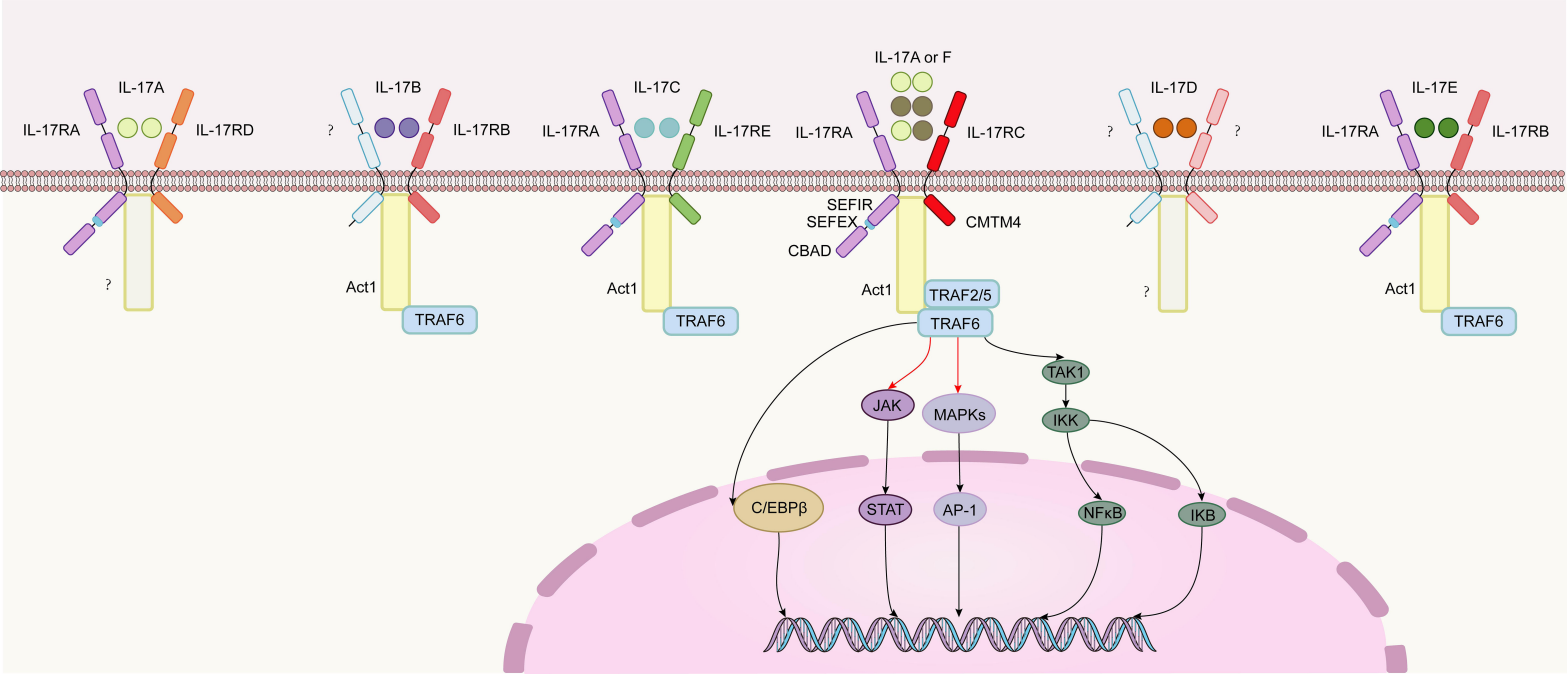
The IL-17 family and signaling pathways: This schematic diagram illustrates the members of the IL-17 cytokine family (IL-17A to IL-17F), their specific receptor pairs on the cell surface, and the subsequent intracellular signaling cascade. The binding of IL-17 ligands to their cognate receptors initiates a downstream pathway involving key adaptor molecules like Act1 and TRAF6, which ultimately activate transcription factors such as NF-κB and AP-1 to drive the expression of pro-inflammatory genes, playing a critical role in immune and inflammatory responses.

Activation of the IL-17R triggers a complex signaling network that involves several key pathways. First, through the adaptor protein TRAF6, the receptor recruits TAK1 kinase and the IKK complex, thereby activating the canonical NF-κB signaling pathway, which drives the expression of numerous inflammatory cytokines and chemokines (39–41). Second, the three principal branches of the MAPK pathway—ERK, JNK, and p38—are sequentially activated, regulating cell proliferation, differentiation, and stress responses (42–44). In certain cell types, activation of the STAT3 pathway is essential for metabolic reprogramming (42). Within these cascades, the adaptor protein Act1 plays a central role. Its unique SEFIR domain interacts with the intracellular segments of IL-17R, ensuring the specificity of signal transduction (45). Recent studies have further demonstrated that IL-17 signaling is finely tuned by multiple negative regulatory mechanisms, including competitive inhibition by soluble receptor variants and post-transcriptional regulation by specific microRNAs (46). These insights have substantially expanded our understanding of the complexity of the IL-17 signaling network and provide a theoretical basis for developing precise therapeutic intervention strategies (**Figure 1**).

Not all IL-17 signaling is created equal. Its outcome hinges on two underappreciated factors—cellular origin and temporal context—which form the backbone of our analysis. We then apply three determinants (injury phase, source, and microenvironment) to explain why IL-17 can be protective in one setting but pathogenic in another.

## 4 Roles of IL-17 across the stages of injury repair

IL-17 exerts complex, context-dependent functions throughout tissue repair, with pronounced stage specificity and environmental sensitivity. Emerging evidence indicates that IL-17 participates in the entire reparative continuum through tightly orchestrated spatiotemporal control, establishing a distinctive dynamic equilibrium across the inflammation–regeneration–remodeling axis.

In the early inflammatory phase, IL-17A from ILC3s, mast cells, and γδ T cells engages fibroblasts and endothelial cells to induce CXCR2 ligands (CXCL1/CXCL8), driving neutrophil and monocyte/macrophage recruitment that supports both pathogen clearance and tissue repair—a sequence consistently observed in lung injury, skin infection, and COPD (2, 47, 48). Beyond its chemotactic role, by directly acting on epithelial cells, IL-17A upregulates pro-survival and growth factors to prime proliferative repair. In keratinocytes, it triggers YAP activation via MST1–ACT1 (relieving MST1–LATS1 inhibition), leading to YAP dephosphorylation, AREG upregulation, and proliferation (49). In fracture repair, IL-17A derived from local γδ T cells not only amplifies the inflammatory response but may also influence systemic inflammatory tone through a proposed “gut–bone axis” mechanism (50). While a calibrated IL-17 response is essential for host defense, excessive production—exemplified by Th17 expansion in diabetic wounds—can drive IL-6/TGF-β-mediated inflammatory dysregulation and ultimately impede repair (51). Thus, IL-17A exerts context-dependent effects on tissue repair—transient augmentation may accelerate regeneration, whereas chronic blockade (e.g., anti-IL-17 biologics) could theoretically delay epithelial healing in the lung or skin.

As repair enters the proliferative phase, IL-17 signaling pivots toward promoting tissue regeneration through several mechanisms (1): it directly activates epithelial programs—such as keratinocyte proliferation and migration—to accelerate re-epithelialization (17) (2); it modulates macrophage polarization, facilitating the M1→M2 transition; in deep-tissue pressure injury (DTPI) models, IL-17A markedly enhances formation of a pro-repair microenvironment by day 7 post-injury (52); and (3) in skeletal repair, it finely tunes osteoprogenitor differentiation, maintaining the dynamic balance between bone formation and resorption (53).

During tissue remodeling, the epigenetic dimensions of IL-17 activity become increasingly evident. IL-17 cooperates with type 2 cytokines (IL-4/IL-13) to coordinate extracellular matrix degradation and reconstruction, shaping disease trajectories across tissues. In tendon ossification, IL-17 influences fibrosis by directing fibroblast differentiation (2). Conversely, sustained IL-17 signaling can drive pathological repair, manifesting as cutaneous fibrosis or even tumor progression (3, 54).

**Figure 2:**
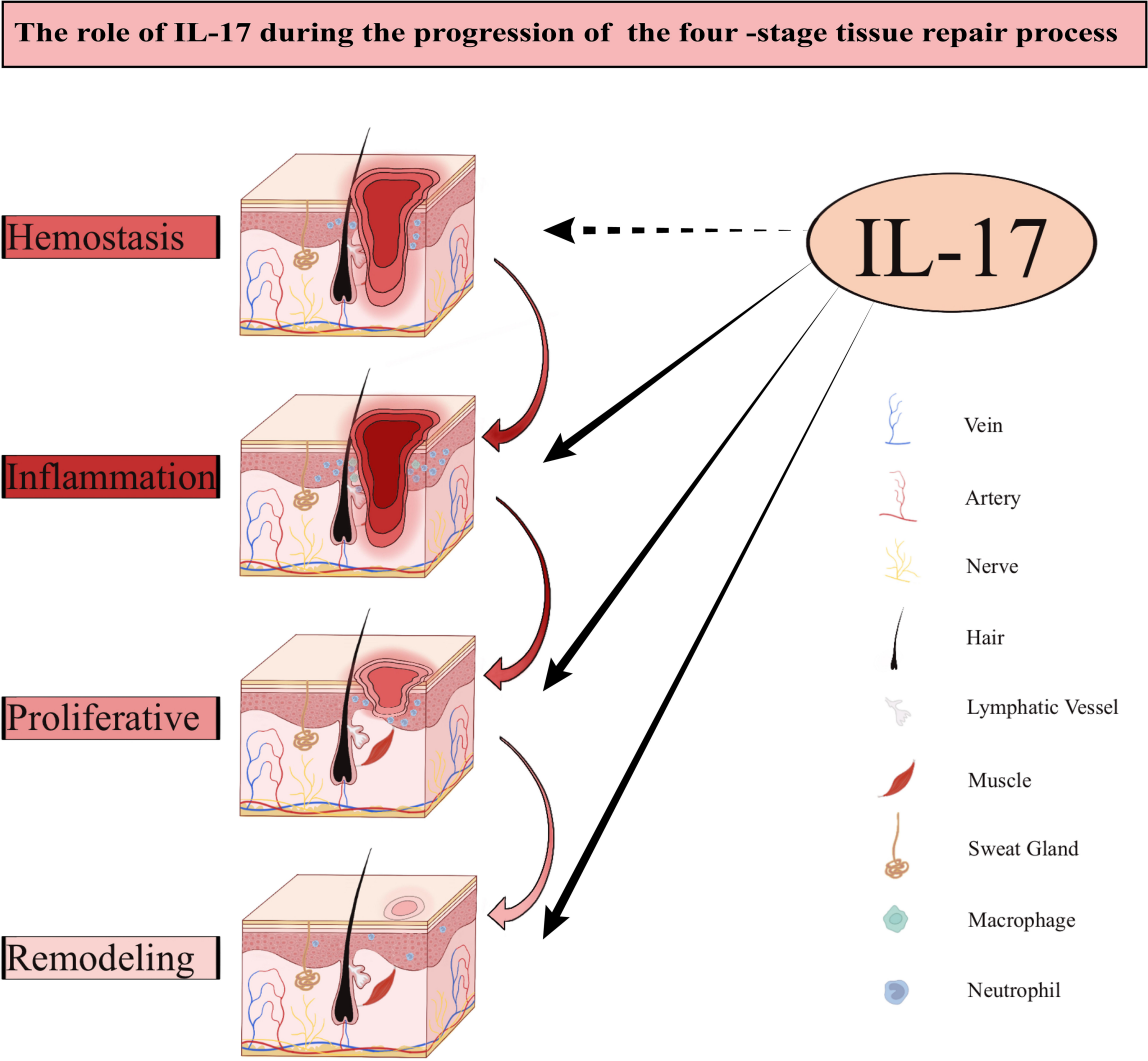
Roles of IL-17 across the stages of injury repair: IL-17 signaling dynamically participates in the four overlapping phases of wound repair. While its role in the hemostasis phase remains unclear (indicated by a dashed arrow), it prominently drives inflammation by recruiting neutrophils and monocytes. During the proliferative phase, IL-17 facilitates re-epithelialization and angiogenesis, while in the remodeling phase, it influences extracellular matrix deposition and scar formation.

**Figure 2** depicts the phase-dependent continuum of IL-17 activity throughout the sequential stages of repair. Nevertheless, under specific circumstances, what determines whether IL-17 exhibits a regenerative versus a pathological trajectory? Based on our integrated analysis of the literature, we propose that the answer resides in a set of key determinants that govern the functional outcome of IL-17 signaling, including (1): the cellular source of IL-17 (innate versus adaptive immune cells) (2); the phase and duration of injury (acute versus chronic) (3); the responding cell population (epithelial versus fibroblast) (4); the strength and persistence of local signaling; and (5) the microenvironmental context (microbial status and the nature of injury — sterile versus infectious). In the following sections, we will apply these determinants uniformly across organ systems to generate a comprehensive insight into when and why IL-17 promotes repair rather than driving fibrosis.

## 5 IL-17’s core mechanism in promoting repair: regulating the “inflammation–proliferation” balance

The dual role of IL-17 in tissue immunity is well known, but its direct contribution to regenerative repair has only been revealed in recent years. Studies across multiple organs indicate that the core function of IL-17 lies in fine-tuning the transition from inflammatory clearance to epithelial proliferation. This section dissects three core mechanisms by which IL-17A mediates this balance in different tissues: metabolic reprogramming, inflammation–proliferation transition, and interactions with the local microenvironment.

### 5.1 IL-17 promotes epithelial regeneration and repair in different tissues

IL-17A binds to its tissue-specific receptor complex (IL-17R), thereby activating downstream signaling networks and initiating a cascade of reparative processes. Recent studies demonstrate that IL-17A precisely regulates epithelial regeneration through three core mechanisms: metabolic reprogramming, inflammation-to-proliferation transition, and microenvironmental interactions. This multi-layered regulatory network allows IL-17A to act in a tissue-specific manner, addressing the distinct regenerative needs of each tissue.

#### 5.1.1 IL-17 in cutaneous repair

In skin repair, acute wounds (e.g., surgical incisions, trauma, burns) require rapid re-epithelialization to restore barrier function, whereas chronic wounds (e.g., diabetic and venous ulcers) are characterized by persistent inflammation and impaired healing. Regarding the cellular sources and responders to IL-17, γδ T cells and RORγT^+^ cells serve as the primary sources in the acute phase, acting on keratinocytes; in the chronic phase and fibrotic diseases (e.g., keloids), the sources shift toward adaptive Th17 cells, which act on fibroblasts (49, 55). Recent multi-omics studies have revealed the pivotal regulatory role of IL-17A in skin injury repair. Consistent with the determinants outlined above, the pro-regenerative function of IL-17 in cutaneous repair is associated with an acute injury phase and innate cellular sources. IL-17A derived from RORγT^+^ cells activates the hypoxia-induced mTOR–HIF1α signaling axis, thereby inducing glycolytic metabolic reprogramming in keratinocytes at the wound edge (5, 18). This metabolic adaptation markedly enhances keratinocyte migratory capacity, accelerating the re-epithelialization process. At the molecular level, IL-17 significantly upregulates proliferation-associated markers such as PCNA, STAT3, and YAP, thereby promoting epidermal regeneration (3, 56). Moreover, ATAC-seq analyses have demonstrated that negative pressure wound therapy (NPWT) markedly alters the chromatin accessibility profile of fibroblasts, with differentially expressed genes (DEGs) significantly enriched in the IL-17 signaling pathway. In rat models, NPWT increased IL-17 expression in wounds, whereas the IL-17 inhibitor secukinumab (SEC) blocked the pro-healing effects of NPWT (57).Recent studies indicate that therapeutic modalities such as fecal microbiota transplantation enhance IL-17 expression within wound tissues and accelerate re-epithelialization through the IL-17/mTOR/HIF-1α signaling axis, thereby promoting repair (58). In contrast, when IL-17 signaling persists and its source shifts to adaptive Th17 cells, it drives pathological processes. Specifically, single-cell RNA-seq of keloids revealed a marked accumulation of Th17 cells, which promote fibroblast proliferation, collagen synthesis, and migration via IL-17A—a mechanism also observed in hypertrophic scars and scleroderma, pointing to a common pathway in skin fibrosis (55, 59). In chronic inflammation and fibrosis, the maladaptive roles of IL-17 are exemplified by excessive IL-17A expression impairing healing in diabetic wounds (51, 60), while in keloids, Th17-derived IL-17 promotes fibrosis via the TGF-β/Smad and STAT3/HIF-1α pathway (61, 62). From a translational perspective, therapeutic strategies must be phase-specific. For acute wounds, innate-derived IL-17 signaling should be preserved or enhanced, as exemplified by negative pressure wound therapy (NPWT), which has been shown to augment IL-17 signaling and promote healing. For chronic wounds and keloids, localized inhibition of Th17-derived IL-17 (e.g., via intralesional injection) may be beneficial, with careful attention to administration route and timing to avoid systemic immunosuppressive risks. Collectively, these findings position IL-17 as a central regulator of cutaneous wound healing, acting through metabolic rewiring and coordinated multi-pathway crosstalk.

#### 5.1.2 IL-17 in pulmonary repair

In pulmonary repair, bleomycin-induced (sterile) and diacetyl-induced (chemical) injuries yield opposite outcomes. In infectious pneumonia, IL-17 is protective early but becomes pathogenic in the chronic phase. γδ T cells dominate in acute injury and early infection, while Th17 cells associate with chronic fibrosis; responder cells include alveolar epithelial cells and fibroblasts. In studies of pulmonary epithelial repair, Tingting Lv and colleagues, based on differential gene expression analysis from single-cell RNA sequencing, found that in AT2 cells following bleomycin (BLM)-induced injury, immune response-related genes such as LCN2 were highly expressed in alveolar epithelial cells. *In vitro* experiments further demonstrated that human alveolar epithelial cells (HPAEpiCs) overexpressing LCN2 exhibited impaired cell viability and proliferation, whereas treatment with recombinant human IL-17 (rhIL-17) partially alleviated LCN2-induced proliferative suppression. Notably, the effect of IL-17 intervention displayed partial dose dependency, with 20 ng/mL rhIL-17 exerting greater restorative effects than 10 ng/mL, while 50 ng/mL did not confer additional benefit (63). Interestingly, IL-17RA-deficient mice exhibited reduced early epithelial injury but delayed later repair. Mechanistic investigations revealed that IL-17 maintains the dynamic balance between inflammatory resolution and proliferative activation through modulation of the LCN2/IL-6 positive feedback loop (64).

A striking contradiction emerges from studies using different lung injury models. In pulmonary fibrosis models, IL-17A neutralization consistently attenuates collagen accumulation and improves lung function (65–67), supporting a pro-fibrotic role. However, in influenza A virus infection, targeting IL-17A alleviates inflammation but has limited effects on lung tissue repair, suggesting that IL-17A may contribute to tissue homeostasis during the post-infection repair phase (68). Zhang H et al. demonstrated that pulmonary commensals such as *Lactobacillus plantarum* and *Lactobacillus murinus* induce IL-17A-mediated antibacterial immunity via Vγ4^+^ γδ T cells, thereby enhancing host resistance to bacterial pneumonia. Conversely, antibiotic treatment reduces the frequency of IL-17A^+^ γδ T cells and increases susceptibility to infection, further reinforcing the protective role of IL-17A in mucosal defense (69). Furthermore, in patients with Mycoplasma pneumoniae pneumonia (MMPP), a reduction in IL-17A+ Th17 cells may impair bacterial clearance capacity, while an increase in the cytotoxic Th17 subset contributes to the hyperinflammatory pulmonary response (70). These apparent contradictions likely reflect not experimental artifacts, but instead the distinct injury models and microenvironments in which IL-17 acts. We propose several non-mutually exclusive explanations for this paradox. The net effect of IL-17 likely depends on the predominant inflammatory driver: in sterile fibrotic models such as bleomycin-induced fibrosis, persistent IL-17 from adaptive Th17 cells promotes fibroblast activation and collagen deposition, whereas in infectious settings (influenza or bacterial pneumonia), IL-17 is primarily produced by innate lymphocytes and γδ T cells in the acute phase, favoring pathogen clearance and tissue repair without chronic fibrotic consequences. Timing matters as well—in influenza infection, IL-17 supports post-repair homeostasis rather than fibrogenesis, suggesting that its pro-fibrotic potential emerges only when inflammation becomes chronic or when Th17 cells dominate. Ultimately, the cellular source may dictate functional outcomes: γδ T cell- and ILC3-derived IL-17 predominates in early mucosal defense and repair, whereas Th17-derived IL-17 is more strongly associated with pathological fibrosis. Neutralization studies that do not distinguish sources may therefore yield seemingly conflicting results across models that differ in their immune cell dynamics, underscoring the need for model-specific and temporally resolved analyses when evaluating IL-17 as a therapeutic target in lung disease.

#### 5.1.3 IL-17 in intestinal epithelial repair

In intestinal repair, the protective role of IL-17 is most evident in the context of microbial challenge and acute injury, where innate sources and barrier integrity mechanisms dominate. In colitis research, histopathological evaluation, analyses of microbial colonization and distribution, and assessments of inflammatory cytokine and lysozyme expression, as well as proximal colonic mucus distribution, were performed in male C57BL/6, BALB/c, Il-10–/–, and Il-17a–/– mice following several weeks of infection with *Helicobacter hepaticus*. The results revealed abnormally high colonic colonization rates in Il-17a–/– mice. Compared with infected C57BL/6 mice, infected Il-17a–/– mice exhibited significantly increased expression of multiple inflammatory genes in the proximal colon, reduced colonic mucus, and downregulated expression of tight junction proteins (ZO-1 and Claudin-1) and IL-22. These findings suggest that IL-17A deficiency impairs intestinal epithelial integrity, diminishes mucus secretion, weakens mucosal regeneration, and reduces resistance to microbial infection, ultimately leading to colitis induced by enteric pathogens (71). In a radiation-induced intestinal injury model, Daqian Huang and colleagues used RNA sequencing and flow cytometry to demonstrate that chondroitin sulfate significantly activated the immune system and upregulated IL-17A and NF-κB signaling, thereby providing protection against radiation-induced intestinal damage (72). In addition, in an *in vitro* model of hyperoxia-induced neonatal intestinal injury, IL-17D was shown to regulate chemokine expression in intestinal epithelial cells under hyperoxic conditions, providing a mechanistic basis for investigating neonatal mucosal immune responses in hyperoxic environments (73).

### 5.2 Promotion of vascular endothelial cell proliferation

An integration of recent studies has demonstrated that IL-17A promotes angiogenesis in diverse tissues through context-specific mechanisms. In a diabetic retinopathy model, histological analyses and *in vitro* assays revealed that IL-17A upregulates VEGF expression, leading to enhanced vascular endothelial cell proliferation, increased vascular branching points, and augmented tubular structures (p < 0.05) (74). These findings suggest that IL-17A may represent a potential therapeutic target for retinal neovascular diseases.

Similarly, in a cerebral ischemia/reperfusion (I/R) injury model, Western blot analysis indicated that effective treatment increased both VEGF protein and mRNA expression in the ischemic penumbra 21 days post-stroke in an IL-17A–dependent manner. This effect was accompanied by co-activation of the JAK2/STAT3 pathway, which significantly elevated vascular density in the penumbral region (p < 0.05). As a result, local perfusion improved, angiogenesis and functional recovery after ischemic injury were promoted, and neuroprotective effects were achieved (75).

### 5.3 Recruitment of reparative immune cells

In studies of acute skeletal muscle injury, flow cytometry analyses revealed that IL-17A–producing γδ T cells rapidly accumulated at the injury site within 24 hours. By secreting IL-17A, these cells recruited CXCR2^+^ reparative neutrophils and pro-regenerative macrophages. In parallel, EdU incorporation assays confirmed that IL-17A directly promoted the proliferation of muscle satellite cells (P < 0.01) (76, 77). In IL-17A^+^ γδ T cell–deficient mice, exogenous supplementation with recombinant IL-17A significantly reversed approximately 80% of the inflammatory defects and 65% of the regenerative impairment (P < 0.01), as validated by histological scoring and muscle strength testing.

**Figure 3:**
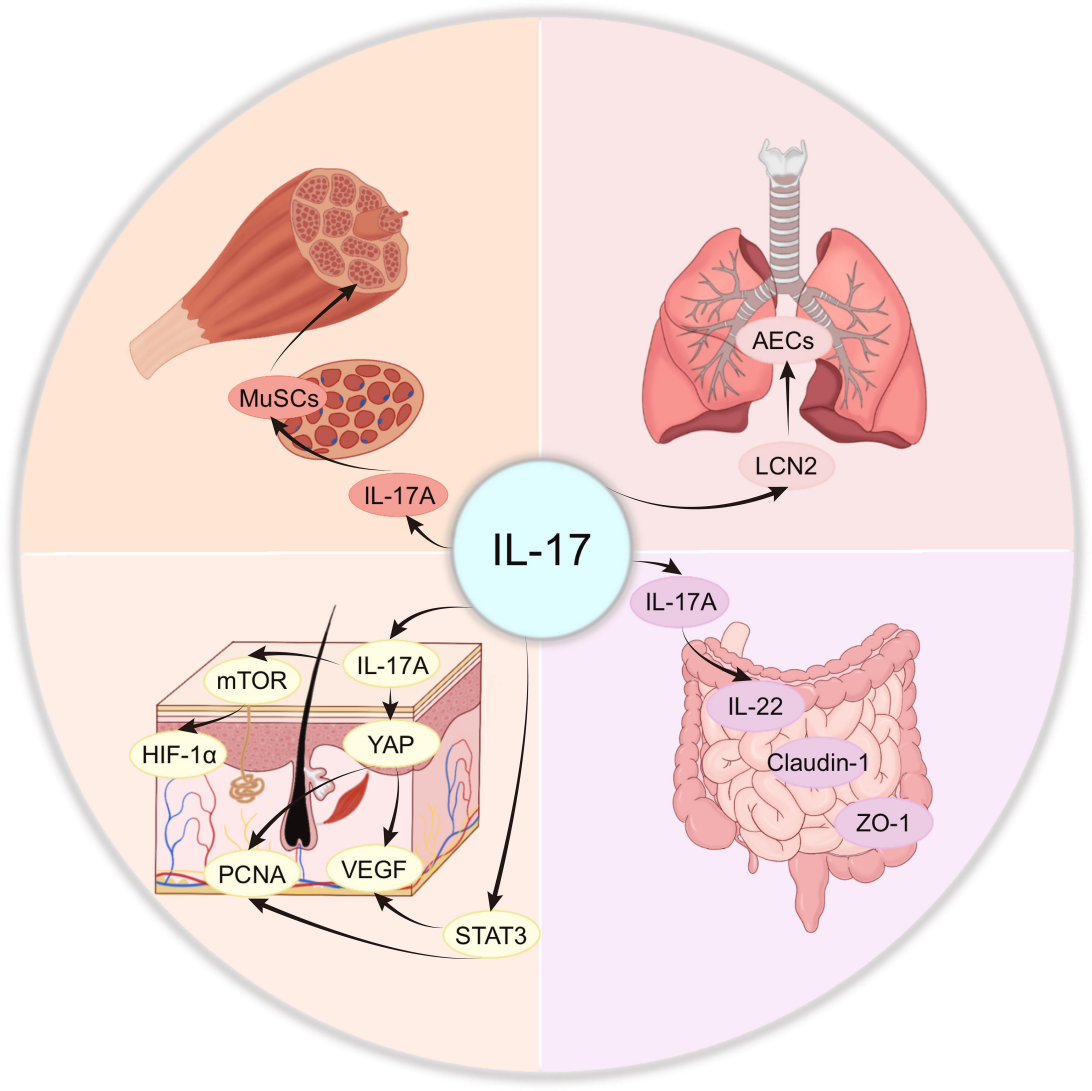
The repair function of IL-17 in different tissues: IL-17 orchestrates tissue-specific repair mechanisms across four major organ systems: in muscle, IL-17A activates muscle satellite cells (MuSCs) to drive regeneration; in the lung, it regulates alveolar epithelial cells (AECs) and the factor LCN2 to balance immune response and repair; in skin, it engages key pathways including mTOR, HIF-1α, STAT3, and YAP to promote keratinocyte proliferation and vascularization; and in the intestine, it synergizes with IL-22 to enhance barrier integrity through tight junction proteins such as Claudin-1 and ZO-1.

Recent findings also indicate that another IL-17 family member, IL-17B, is highly expressed in regenerating muscle fibers of patients with Duchenne muscular dystrophy (DMD), suggesting its potential as a novel biomarker of muscle regeneration (78). Moreover, a study published by Professor Zhengxin Ying’s group in Cell Reports demonstrated that Schwann cells promote macrophage recruitment and axonal regeneration after nerve injury through the IL-17B/IL-17RB signaling pathway, thereby facilitating neural repair (31) (**Table 1**, **Figure 3**).

**Table 1:** Summary of the functions and molecular mechanisms of IL-17 in multitissue.

Functional role	Tissue/model	Key mechanisms	Major findings	References
Epithelial regeneration	Skin repair	mTOR–HIF1α axis–mediated glycolytic reprogramming; upregulation of PCNA, STAT3, YAP	Enhances keratinocyte migration; accelerates re-epithelialization; promotes epidermal regeneration via multiple pathways	(3, 5, 18, 56)
	Lung repair	Regulation of the LCN2/IL-6 positive feedback loop; macrophage M2 polarization	Reverses LCN2-induced inhibition of alveolar epithelial proliferation; IL-17RA deficiency delays repair; paradoxically, IL-17A neutralization aggravates pulmonary fibrosis	(63, 64)
	Intestinal repair	Regulation of tight junction proteins (ZO-1, Claudin-1); induction of MUC2 secretion; microbiota–Treg interactions	IL-17A deficiency impairs barrier integrity; commensal-derived SCFAs promote Treg differentiation and suppress excessive inflammation	(71, 84, 85)
	Liver repair		No direct evidence	–
Angiogenesis	Diabetic retinopathy	Upregulation of VEGF	Increases endothelial cell proliferation, vascular branching, and tubular structures	(26, 27)
	Cerebral ischemia	IL-17A–dependent activation of JAK2/STAT3 pathway	Enhances vascular density in the ischemic penumbra; improves local perfusion and angiogenesis	(75, 105)
Recruitment of reparative immune cells	Skeletal muscle injury	γδ T cell–derived IL-17A recruits CXCR2^+^ neutrophils and macrophages; activation of satellite cell proliferation	γδ T cells rapidly accumulate post-injury; recombinant IL-17A rescues ~80% of inflammatory defects and ~65% of regenerative impairment	(76, 77)

## 6 Key mechanisms of the pathological effects of IL-17: disruption of homeostasis repair

IL-17 exerts its pathological effects largely by disrupting the normal repair homeostasis of tissues. This disruption occurs through two major mechanisms: first, IL-17 amplifies local inflammatory responses, leading to sustained tissue damage and barrier dysfunction; second, it drives pathological fibrosis, promoting excessive extracellular matrix deposition and organ scarring. Together, these mechanisms shift the tissue environment from regenerative repair toward chronic injury and dysfunction.

### 6.1 IL-17–mediated amplification of local inflammation

In localized inflammatory amplification, psoriasis, pneumonia, and inflammatory bowel disease represent distinct pathological contexts where IL-17 drives inflammation, but the determinants of IL-17 signaling outcomes vary. In chronic inflammation, Th17 cells are the main source of IL-17, with tissue-specific responder cells: keratinocytes and macrophages in skin, alveolar epithelial cells and neutrophils in lung, and intestinal epithelial cells and lamina propria immune cells in gut. Current evidence indicates that IL-17A exerts potent pro-inflammatory effects across multiple tissues through a three-tiered signaling cascade. This mechanism involves three critical steps: first, IL-17A markedly increases the phosphorylation of NF-κB and p38 MAPK, thereby activating downstream signaling pathways; second, this activation drives the robust release of pro-inflammatory cytokines such as IL-6, TNF-α, and IL-1β; and third, these events culminate in tissue structural damage, manifested as barrier dysfunction and inflammatory cell infiltration.

In inflammatory skin diseases such as psoriasis, IL-17 and the IL-23/IL-17A axis play a central pathogenic role. Specifically, dendritic cells in the skin produce IL-23, which activates the IL-23/IL-17A cytokine axis. Through the Act1/TRAF6/TAK1/NF-κB pathway, this activation promotes inflammatory responses in keratinocytes and macrophages, thereby intensifying cutaneous inflammation and ultimately driving the severe desquamation characteristic of psoriasis (79, 80). In addition, IL-17 enhances keratinocyte proliferation via STAT3 and p38 activation and upregulates pro-inflammatory mediators, including IL-1β, IL-6, and IL-22, further aggravating psoriatic dermatitis (81, 82). A similar mechanism has been implicated in cutaneous wound disorders such as diabetic ulcers. In diabetic wounds, excessive IL-17A expression impairs tissue repair, in part due to the expansion of Th17 CD4^+^ cells. These cells secrete excessive IL-6 and TGF-β, reinforcing the Th17 inflammatory phenotype. Consequently, local inflammation is exacerbated and extracellular matrix dynamics are disrupted, collectively suppressing wound healing (51).

In pulmonary infectious diseases such as pneumonia, IL-17 promotes the expression of chemokines, including CXCL1 and CXCL5, leading to massive neutrophil infiltration into lung tissue. These infiltrating neutrophils release elastase and ROS, disrupting redox homeostasis and directly causing alveolar epithelial injury and structural damage to lung tissue (83). More complex still, IL-17 enhances the production of pro-inflammatory cytokines such as TNF-α and IL-6 in lung tissue or plasma, thereby amplifying the inflammatory response through synergistic effects. Intriguingly, these changes are accompanied by altered downstream IL-17R signaling via the IL-17R/Act1/TRAF6/IκBα pathway, which shifts immune cell recruitment and polarization from anti-inflammatory to pro-inflammatory states, ultimately exacerbating lung injury and worsening pneumonia (83). Moreover, a high-calorie diet disrupts the intestinal microbiota structure, reduces short-chain fatty acid (SCFA) levels, alters the Th17/Treg balance, and exacerbates LPS-induced pulmonary inflammatory damage (84, 85).

In the context of intestinal inflammation, studies have demonstrated that IL-17A levels are significantly elevated in the duodenal mucosa of patients with psoriasis, and this elevation correlates directly with gastrointestinal symptoms such as abdominal pain and diarrhea (86). Mechanistically, IL-23 drives Th17 cell differentiation and IL-17 secretion, thereby promoting the infiltration of neutrophils and monocytes into the intestinal lamina propria and contributing to the progression of ulcerative colitis (87, 88). However, the role of IL-17 in intestinal inflammation is far more complex than its pro-inflammatory properties alone would suggest. For example, although Th17 cells and IL-17A are widely regarded as major pathogenic factors in inflammatory bowel disease (IBD), several studies have unexpectedly revealed that IL-17A-neutralizing antibodies—theoretically designed as a therapeutic strategy for IBD—not only fail to improve the disease but may instead exacerbate intestinal inflammation (89–91). Meanwhile, members of the IL-17 family exhibit distinct functions: IL-17B has been demonstrated to exert protective effects against colitis, IL-25 (IL-17E), secreted by intestinal epithelial cells, participates in regulating type 2 immunity and mucosal repair (92, 93). This finding carries significant clinical implications, suggesting that a broad-spectrum neutralization strategy may inadvertently target protective subtypes, and the use of IL-17 inhibitors such as ixekizumab may disrupt IL-17–mediated regulation of intestinal homeostasis and thereby aggravate IBD pathology in susceptible individuals.

How can this intestinal paradox be explained? Recognizing that IL-17A plays a non-redundant role in maintaining epithelial barrier integrity, promoting antimicrobial peptide production, and supporting mucosal repair—functions that are disrupted by broad neutralization. Moreover, the cellular source of IL-17 is critical: ILC3-derived IL-17 is largely protective, whereas Th17-derived IL-17 can drive pathology—a distinction that neutralizing antibodies cannot discriminate. The failure of IL-17 inhibitors in IBD serves as a cautionary example: therapeutic efficacy depends not merely on the presence of IL-17, but on the dominant local determinants—particularly barrier integrity and cellular source. Thus, the intestinal paradox underscores that IL-17’s net effect reflects a dynamic balance between barrier protection and inflammatory pathology, rather than a fixed pro- or anti-inflammatory label.

### 6.2 IL-17 as a driver of pathological fibrosis

In fibrosis, keloids, pulmonary fibrosis, and liver cirrhosis are distinct diseases where IL-17 drives excessive extracellular matrix deposition, all involving chronic injury and persistent inflammation. Th17 cells are the main source of IL-17 in these fibrotic conditions, acting on fibroblasts (skin, lung) and hepatic stellate cells (liver). Current evidence suggests that excessive IL-17 expression during the chronic phase of injury repair promotes fibrosis through multiple mechanisms (1): direct activation of fibroblast functions (2); modulation of key fibrogenic signaling pathways (e.g., TGF-β/Smad and STAT3) (3); regulation of autophagy and cell death processes; and (4) disruption of the balance between pro-fibrotic and anti-fibrotic immune responses. Notably, these mechanisms exhibit marked tissue specificity.

In contrast to the acute regenerative context, the fibrotic role of IL-17 in keloids illustrates how shifts in the key determinants can transform the cytokine’s function. Consistent with the importance of cellular source, recent single-cell transcriptomic analyses have revealed that the proportion of Th17 cells—rather than γδ T cells—is significantly elevated in keloid tissue compared with normal scar tissue (55). These Th17-derived IL-17A signals directly stimulate keloid fibroblast proliferation, collagen synthesis, and migration. Mechanistically, this IL-17 promotes fibrosis by upregulating SDF-1 (stromal cell–derived factor-1) and HIF-1α (hypoxia-inducible factor-1α) pathways, thereby enhancing the expression of fibrotic markers such as collagen and α-SMA. In parallel, IL-17 augments fibroblast activity through activation of the TGF-β/Smad pathway and induces autophagy dysfunction via STAT3/HIF-1α signaling (94, 95). Here, the responder cell population has shifted from keratinocytes to fibroblasts, the signaling is chronic and sustained, and the inflammatory microenvironment is dysregulated—all factors that favor fibrosis over regeneration.

In addition, IL-17 has been linked to the transcriptional regulation of TRAF3IP2 (TRAF3-interacting protein 2), which amplifies inflammatory responses via downstream NF-κB signaling, further contributing to keloid pathogenesis (96).

In pulmonary fibrosis, IL-17 exacerbates tissue scarring by inhibiting autophagy, a protective anti-fibrotic mechanism, while simultaneously upregulating hydroxyproline content and collagen deposition through a TGF-β–dependent pathway (97, 98). Multiple studies further demonstrate that elevated IL-17A levels correlate with reduced lung function, and IL-17 blockade attenuates collagen accumulation in BLM or silica-induced models of pulmonary fibrosis (65, 99, 100). However, a critical contradiction challenges this pro-fibrotic model: Moog et al. (101) demonstrated that Il17a/f knockout mice still develop significant collagen deposition and structural remodeling after BLM exposure, comparable to wild-type controls. This finding suggests that while IL-17 can contribute to fibrosis, it is not universally indispensable for fibrotic progression. How can this apparent contradiction be resolved? The most likely explanation is cytokine redundancy—other pro-fibrotic mediators such as TGF-β1, IL-13, IL-11, and IL-33 can drive fibrosis through parallel pathways when IL-17 signaling is absent (102, 103). Additionally, the dominant inflammatory microenvironment matters: models with a strong Th17 signature show greater dependency on IL-17, whereas those driven by Th2-biased responses may not. The temporal dimension is also key—as discussed earlier, IL-17 may be protective in acute injury but pathogenic during chronic remodeling, and constitutive knockout models cannot capture this duality. Finally, cellular source likely contributes: γδ T cell-derived IL-17 can be protective in acute injury, while Th17-derived IL-17 drives chronic fibrosis. Thus, IL-17 acts more as an amplifier than an essential initiator of pulmonary fibrosis. Its therapeutic inhibition is most beneficial in diseases where it is the dominant driver, rather than in settings where multiple parallel pathways are activated—a nuance with important implications for patient selection and combination therapy in clinical trials.

**Figure 4:**
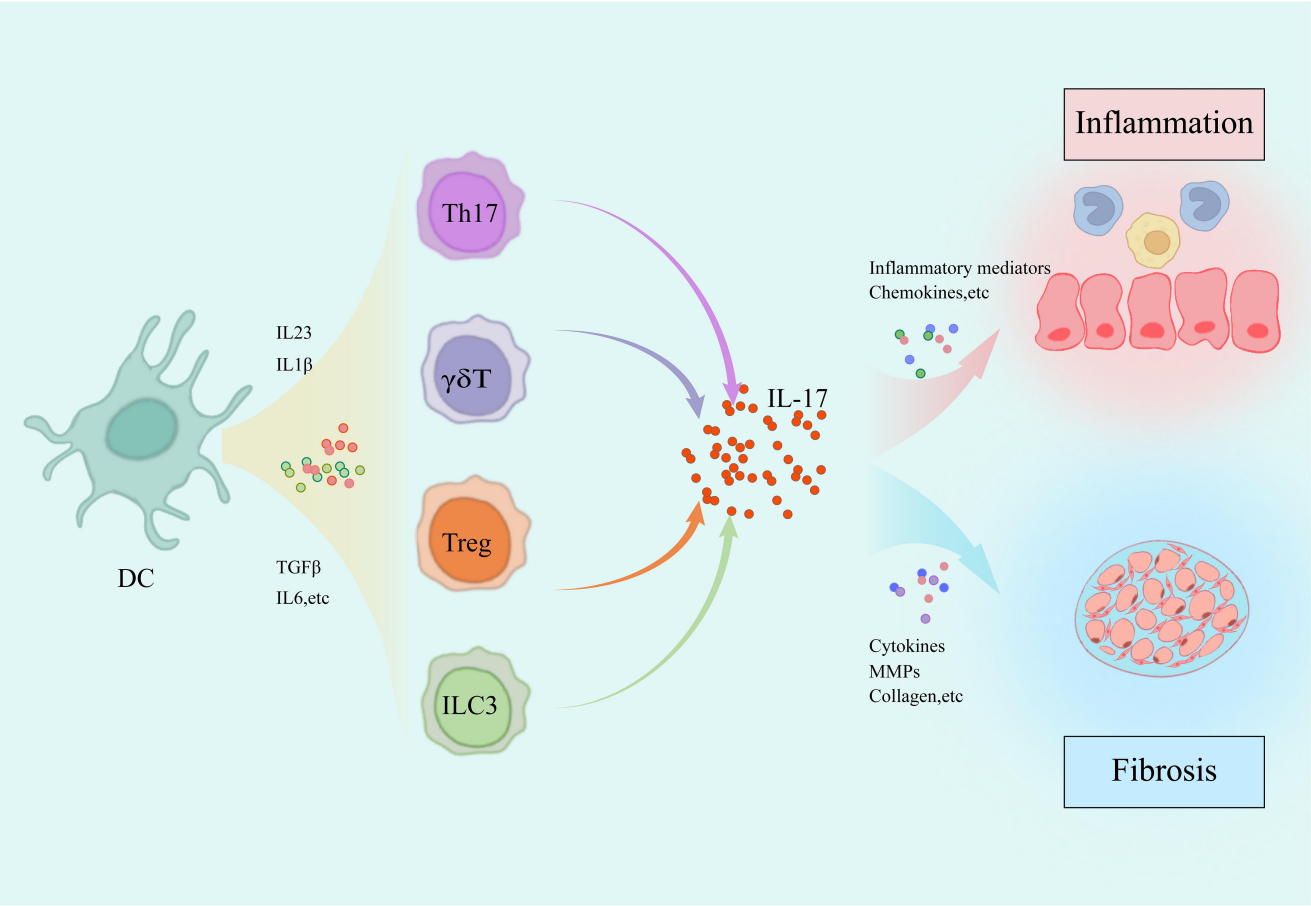
Cellular and molecular mechanisms underlying IL-17-driven pathological effects: Dendritic cells (DCs) secrete polarizing cytokines (IL-23, IL-1β, TGF-β, and IL-6) that drive the differentiation and activation of adaptive (Th17) and innate (γδ T cells, ILC3) IL-17-producing cells, while modulating regulatory T (Treg) cell function; excessive IL-17 signaling bifurcates into dual pathological outcomes—amplification of local inflammation via induction of inflammatory mediators and chemokines (e.g., CXCL1, CXCL5, IL-6, TNF-α) that recruit neutrophils and mononuclear phagocytes, leading to epithelial barrier disruption and oxidative tissue damage; and promotion of pathological fibrosis via direct activation of fibroblasts or hepatic stellate cells to secrete matrix metalloproteinases (MMPs), collagen, and pro-fibrotic cytokines, resulting in excessive extracellular matrix deposition and scar formation. These convergent mechanisms shift the tissue microenvironment from regenerative repair toward chronic inflammation and fibrotic remodeling.

In liver fibrosis, IL-17A acts directly on hepatic stellate cells (HSCs), upregulating pro-fibrotic genes such as α-SMA and collagen, thereby promoting HSC activation and fibrogenesis. Mechanistic studies indicate that IL-17A enhances HSC profibrotic activity by activating the JUN pathway and TGF-β receptor II signaling (104). Additionally, IL-17A may exacerbate fibrosis by inducing IL-6 expression, which activates the JAK2/STAT3 pathway and further augments HSC activation (105). From an immunological perspective, IL-17A fosters Th17 differentiation while suppressing regulatory T cell (Treg) function, thereby disrupting the Th17/Treg balance in favor of pro-fibrotic immunity. For example, in patients with chronic hepatitis B (CHB), HBeAg promotes Th17 differentiation and IL-17A secretion via M1 macrophages, thereby accelerating hepatic fibrosis (106, 107). From a translational perspective, the contradictory findings suggest that IL-17 acts as an amplifier rather than an essential initiator of fibrosis. Therefore, IL-17 inhibition may have therapeutic value only in diseases where it serves as the dominant driver, whereas its efficacy may be limited in conditions where multiple parallel pathways are activated. This has important implications for patient selection and combination therapy strategies in clinical trials (**Table 2**, **Figure 4**).

**Table 2:** Overview of the mechanisms by which IL-17 mediates inflammatory injury and tissue fibrosis.

Functional type	Tissue/model	Key molecular Mechanisms	Major pathological features	References
Excessive inflammation	Skin (psoriasis, diabetic wounds)	IL-23/IL-17A positive feedback loop; STAT3/p38 activation; chemokine and pro-inflammatory cytokine release	Abnormal epidermal hyperplasia; neutrophil and monocyte infiltration	(81, 82)
	Lung (pneumonia)	CXCL1/CXCL5-mediated neutrophil recruitment; synergistic activation of NF-κB/MAPK with TNF-α and IL-6	Alveolar epithelial injury; structural damage to lung tissue	(83, 84)
	Intestine (IBD)	IL-23–driven Th17 differentiation; neutrophil and monocyte infiltration	Elevated IL-17A in duodenal mucosa; abdominal pain and diarrhea	(89–91)
Tissue fibrosis	Skin (keloid scars)	Upregulation of SDF-1/HIF-1α; TGF-β/Smad activation; STAT3/HIF-1α–induced autophagy defects	Increased collagen and α-SMA expression; fibroblast proliferation and migration	(94, 95)
	Lung fibrosis	Autophagy inhibition; TGF-β–dependent collagen deposition	Increased hydroxyproline; impaired lung function	(61, 62)
	Liver fibrosis	JUN/TGF-βRII pathway activation; JAK2/STAT3 activation; Th17/Treg imbalance	HSC activation (α-SMA↑); collagen deposition	(105–107)

## 7 Determinants of the bidirectional effects of IL-17

**Figure 5:**
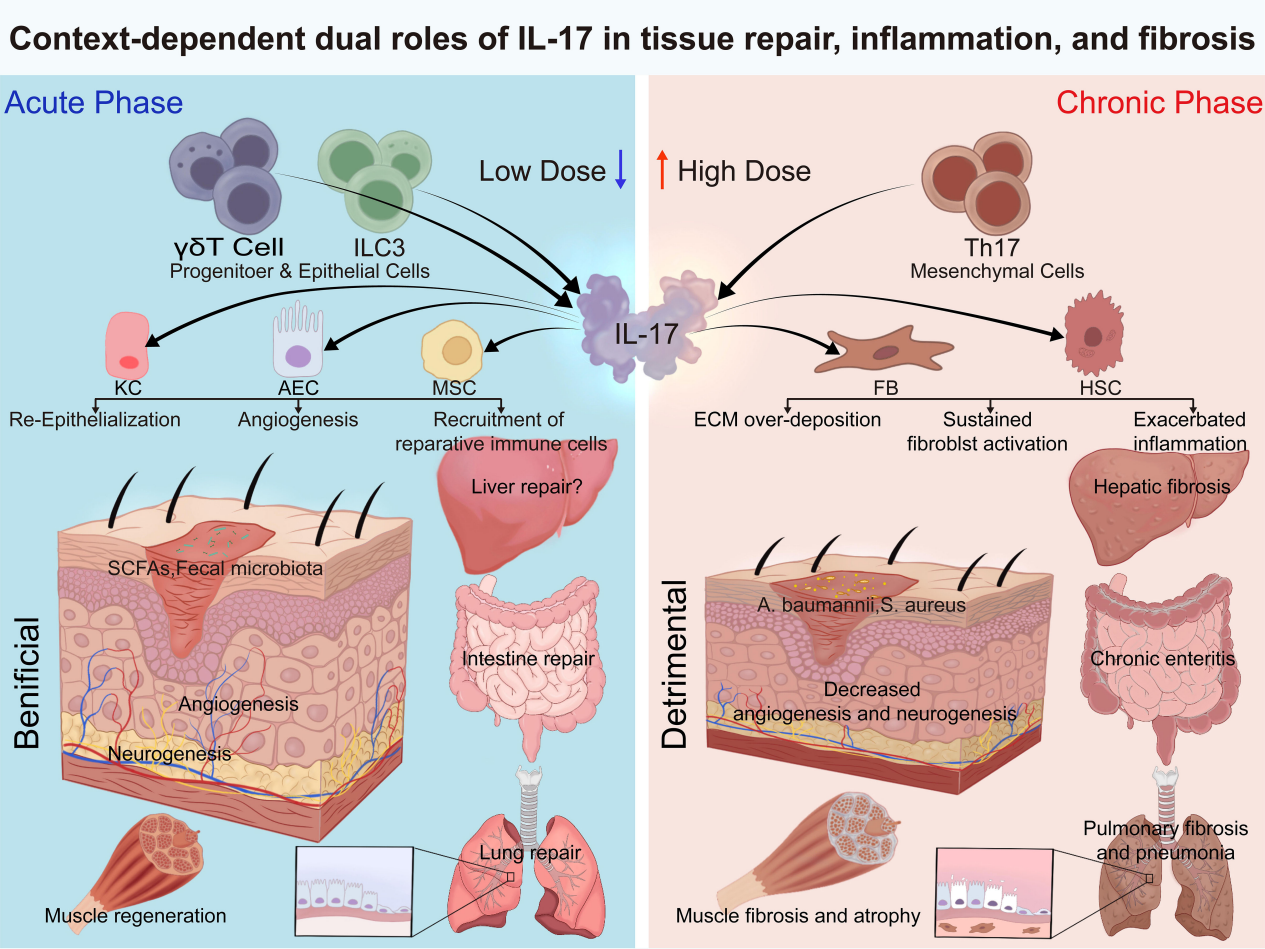
Context-dependent dual roles of IL-17 in tissue repair, inflammation, and fibrosis: IL-17 exerts bidirectional functions governed by spatiotemporal determinants and cellular sources across tissue microenvironments: in acute injury and infectious settings, innate-derived IL-17 from γδ T cells and ILC3s promotes tissue repair by driving epithelial regeneration, angiogenesis, and recruitment of reparative immune cells; in chronic sterile inflammation and fibrotic disease, adaptive Th17-derived IL-17 drives pathological remodeling through sustained NF-κB/MAPK activation, neutrophil-mediated tissue damage, and fibroblast activation with excessive collagen deposition; and the functional balance between protection and pathology is dictated by key determinants including cellular origin (innate versus adaptive), injury phase (acute versus chronic), local signaling intensity, and microbial context.

The biological effects of IL-17 exhibit pronounced bidirectionality across different organ systems. The apparent contradictions—where IL-17 neutralization succeeds in some disease models but fails or even exacerbates pathology in others—are not experimental artifacts but reflect genuine biological complexity. Four recurring themes help explain when IL-17 becomes protective versus pathogenic: the nature of injury (sterile versus infectious), the cellular source (innate versus adaptive), the timing of signaling (acute versus chronic phase), and cytokine network redundancy. These determinants do not operate in isolation. A deeper understanding of them—including microbial modulation, concentration dependency, and temporal dynamics—is critical for developing context-specific therapeutic strategies (**Figure 5**).

### 7.1 Influence of microbial regulation on the bidirectional effects of IL-17

The interaction between the microbiota and the IL-17 signaling pathway is highly complex. Studies have shown that intestinal dysbiosis can activate the IL-17/IL-17RA signaling axis, thereby disrupting local intestinal immune homeostasis and even promoting the growth of distal tumors (108).

As the primary source of IL-17, Th17 cells are tightly regulated by intestinal microbes and their metabolites. Specific bacterial taxa can secrete cytokines that induce Th17 differentiation, and these cells subsequently shape the mucosal immune microenvironment by releasing IL-17, IL-21, and IL-26. In recent years, fecal microbiota transplantation has achieved notable progress in the treatment of IBD, with preliminary evidence supporting its safety and efficacy. These findings highlight microbiome-targeted manipulation as a promising strategy for intervening in IL-17–related diseases (109, 110). At the molecular level, microbial metabolites such as short-chain fatty acids (SCFAs) regulate IL-17 expression through the mTORC1–HIF1α signaling pathway, exerting either inhibitory or promoting effects on inflammation (111). A similar regulatory mechanism has been further validated in diabetic wound models: FMT was shown to activate the IL-17A/mTOR/HIF1α axis, paradoxically enhancing wound healing in diabetic mice (58).

### 7.2 Impact of concentration gradients on IL-17 function

The biological effects of IL-17 display a clear concentration gradient. At low concentrations, IL-17 exerts protective effects by inducing the expression of antimicrobial peptides, such as lipocalin-2 (LCN2), thereby effectively suppressing pathogen proliferation. This mechanism is particularly important in maintaining mucosal immune defense. However, when IL-17 levels exceed a critical threshold, its biological functions shift dramatically. High concentrations of IL-17 can trigger excessive inflammatory responses, leading to pathological alterations such as acute lung injury (112). For example, in an LCN2-induced lung epithelial cell injury model, treatment with 20 ng/mL rhIL-17 showed stronger protective effects compared with 10 ng/mL, whereas 50 ng/mL rhIL-17 failed to confer additional benefit (63). Similarly, Chansu Lee and colleagues, using a human small intestinal enteroid model, demonstrated that IL-17 impaired enteroid formation efficiency, reduced cell viability, and facilitated Enterobacteriaceae overgrowth in a dose-dependent manner (90). These observations underscore the biphasic, concentration-dependent effects of IL-17. Clinically, they highlight the necessity of precisely regulating IL-17 levels: maintaining its protective role in host defense while avoiding pathological tissue damage induced by excessive activation.

### 7.3 Functional differences of IL-17 in acute versus chronic inflammation

IL-17 exerts markedly distinct biological functions in acute and chronic inflammatory settings. During acute injury—such as cutaneous or skeletal muscle trauma—IL-17 accelerates tissue repair by promoting the proliferation of keratinocytes or muscle satellite cells, thereby enhancing wound closure and muscle regeneration (3, 5, 18, 76). For example, in the early phase of skeletal muscle injury, IL-17 significantly upregulates satellite cell proliferation and facilitates muscle regeneration (76). Furthermore, in a study of muscular dystrophy, IL-17B was identified as a potential novel biomarker of human muscle regeneration in degenerative muscle diseases (77). In contrast, under conditions of chronic, persistent inflammation, IL-17 undergoes a functional shift. Instead of supporting regeneration, it amplifies inflammatory responses, suppresses muscle repair, and promotes muscle atrophy (113, 114).

In cutaneous injury, similar mechanisms have been described. During the acute phase, RORγT^+^ cells at the wound edge rapidly respond to hypoxic stress, markedly upregulating IL-17. By binding to its receptors on keratinocytes, IL-17 promotes re-epithelialization and accelerates wound closure. In contrast, in the chronic phase, IL-17 contributes to tissue pathology via two major mechanisms (1): activating multiple signaling pathways, including CEBP, to promote tissue fibrosis and potentially tumor progression; and (2) amplifying inflammatory responses, thereby enhancing leukocyte infiltration and delaying healing of chronic or infected wounds (115). This functional switch may be linked to long-term alterations in the inflammatory microenvironment.

In respiratory diseases, the temporal divergence of IL-17’s functions is even more evident. In the early stages of infection, IL-17 induces cytokine and chemokine production, driving immune cell recruitment to infection sites and enhancing pathogen clearance (116, 117). However, persistent IL-17 signaling in chronic phases contributes to the progression of conditions such as chronic obstructive pulmonary disease (COPD), lung cancer, cystic fibrosis, and asthma (47, 118). This bidirectional role underscores the need to tailor therapeutic strategies according to disease stage, while also offering critical insights for the future development of vaccines against respiratory diseases.

## 8 Therapeutic potential and challenges of targeting IL-17

IL-17 is a well-established driver of several autoimmune diseases, including psoriasis, psoriatic arthritis, axial spondyloarthritis, rheumatoid arthritis, and multiple sclerosis (119–122). Clinical trials have confirmed that monoclonal antibodies targeting IL-17 (e.g., secukinumab, ixekizumab) or its receptor (e.g., brodalumab) are effective in these conditions, and dual inhibition of IL-17A and IL-17F may offer further therapeutic advantages (123–126).

Beyond autoimmunity, IL-17 blockade has shown promise in oncology (e.g., bladder cancer), diabetic retinopathy, and cardiovascular or neurodegenerative diseases (127–131). However, the success in autoimmunity does not automatically translate to tissue repair. As noted in Section 5.1, IL-17 inhibitors can paradoxically exacerbate inflammatory bowel disease (90) underscoring that therapeutic outcomes are highly context-dependent.

When applied to tissue repair, IL-17-targeted therapy faces distinct challenges. Long-term systemic inhibition increases susceptibility to fungal and bacterial infections—a risk particularly concerning in chronic wounds, which are already prone to infection. Localized delivery strategies can mitigate this risk by concentrating the drug at the wound site while preserving systemic immunity, though careful design is required to avoid impairing local immune surveillance. The dual roles of IL-17—protective in acute wound healing and intestinal barrier repair, but pathogenic in chronic fibrosis—preclude a one-size-fits-all approach. Selective targeting of pathological Th17-derived IL-17 while preserving protective innate-derived IL-17 remains an urgent priority, potentially requiring source-specific biomarkers (5, 132). The failure of IL-17 inhibitors in IBD serves as a cautionary example: a therapy effective in psoriasis may be ineffective or harmful in another context depending on the dominant determinants (90). Regarding IL-17F, although it shares partial redundancy with IL-17A, their contributions differ across tissues. Dual inhibition may offer superior efficacy in some autoimmune settings, but whether this holds true for tissue repair—and whether it increases infection risk—requires further investigation (123, 133, 134). Current biologics are costly, require parenteral administration, and are not optimized for tissue repair applications. Expanding delivery routes (e.g., localized injection, transdermal systems, microneedle patches, inhaled aerosols) and developing small-molecule inhibitors would improve applicability (135–137).The therapeutic potential of IL-17 modulation in tissue repair is still emerging, with possible roles in neuroinflammation (e.g., glaucoma), urological malignancies, and cardiovascular diseases (138, 139). However, given the context-dependent biology highlighted throughout this review, each new application demands rigorous preclinical evaluation. Finally, IL-17A acts in concert with pathways such as IL-23 and TNF-α; combination regimens may be beneficial but must be carefully balanced to avoid over-immunosuppression (140).

In summary, striking a balance between the beneficial and pathological effects of IL-17 remains the central challenge. Future priorities include optimizing treatment timing, individualizing therapy based on dominant determinants, and advancing localized delivery systems. The contradictory findings discussed throughout this review serve as important guardrails: therapeutic strategies must be grounded in a nuanced understanding of specific biological contexts rather than one-size-fits-all assumptions.

## 9 Conclusion

IL-17 exerts bidirectional functions in tissue injury repair, acting as both a pro-inflammatory mediator and a pro-regenerative factor. Its effects are highly tissue-specific and finely regulated by factors such as the microbiome, concentration gradients, and the temporal context of acute versus chronic inflammation. This review moves beyond descriptive cataloging to provide an integrated framework centered on key determinants—cellular source (innate versus adaptive), injury phase (acute versus chronic), responder cells (epithelial versus fibroblast), signaling intensity, and microenvironment. By organizing the literature around these determinants, we clarify when and why IL-17 promotes regeneration rather than fibrosis. Apparent contradictions—such as the protective versus pathogenic roles in different lung injury models or the failure of IL-17 inhibitors in IBD despite success in psoriasis—reflect genuine biological complexity rather than artifacts. These inconsistencies are reconciled by recurring principles: injury nature (sterile versus infectious), cellular source, signaling timing, and cytokine network redundancy.

Translational implications follow directly. For acute injuries, preserve or enhance innate-derived IL-17 signaling (e.g., NPWT, biomaterials). For chronic wounds and fibrotic diseases, localized inhibition of Th17-derived IL-17 (intralesional, topical, inhaled) may be beneficial, with attention to route, timing, and combination therapy. The paradoxical failures underscore the need for patient selection and context-aware trial design. Ultimately, expanding IL-17-targeted interventions from autoimmunity to tissue repair holds promise, provided strategies are context-specific. Future priorities include optimizing timing, advancing localized delivery, and developing source-specific biomarkers.
